# Gluten-Free Diet and Health-Related Quality of Life: The Validated Hellenic Version of the Celiac Dietary Adherence Test

**DOI:** 10.3390/nu17020353

**Published:** 2025-01-20

**Authors:** Emmanuel Psylinakis, Alexios Manidis, Fotios Makris, Nikolaos Thalassinos, Anastasia Markaki, Vasileia Kounelaki, Eirini Sfakianaki, Aspasia Spyridaki

**Affiliations:** 1 Department of Nutrition and Dietetics Sciences, Hellenic Mediterranean University (HMU), Trypitos Area, 72300 Sitia, Greece; psylinak@hmu.gr (E.P.); myx47@edu.hmu.gr (A.M.); yd20371@edu.hmu.gr (F.M.); thalassinos@hmu.gr (N.T.); anmarkaki@hmu.gr (A.M.); kounelaki-vasileia@outlook.com (V.K.); sfakianaki@hmu.gr (E.S.); 2 Institute of Agri-Food and Life Sciences, Hellenic Mediterranean University Research Centre, 71410 Heraklion, Greece

**Keywords:** celiac disease, gluten-free diet, dietary adherence, celiac dietary adherence test, health-related quality of life, validation, nutrition

## Abstract

Background/Objectives: A reliable assessment of gluten-free diet (GFD) adherence is essential for managing celiac disease (CD). This study aimed to validate the Hellenic version of the Celiac Disease Adherence Test (H-CDAT) to evaluate adherence levels and explore the impact of dietary adherence on health-related quality of life (HRQoL)—both of which have never been objectively assessed in Greek CD patients. Methods: The study included 102 adult CD patients who completed H-CDAT, diet-related questions, and the 36-Item Short Form Health Survey (SF-36). Results: H-CDAT demonstrated good psychometric properties and showed multiple strong correlations with HRQoL dimensions. H-CDAT adherence was Good in 38.2%, Moderate in 42.2%, and Poor in 19.6% of patients, despite their perception of effective adherence, with 51% never having visited a dietitian for guidance on a GFD. Patients scored significantly lower across most HRQoL dimensions compared to the general Greek population. When stratified into the three adherence categories, striking differences emerged between Good and Moderate adherence across both physical and mental health domains, highlighting that moderate adherence is not sufficient for optimal health outcomes. Conclusions: These findings emphasize the critical role of strict GFD adherence in improving overall health and underscore the importance of dietetic intervention for achieving optimal patient outcomes.

## 1. Introduction

Celiac disease (CD) is a serious chronic autoimmune disorder that affects genetically susceptible individuals, where gluten ingestion triggers an immune response, leading to intestinal inflammation and damage [1]. The prevalence of CD in Europe is estimated to range from 1% to 2% of the population [2].

Classic CD symptoms include gastrointestinal issues such as diarrhea, abdominal pain, bloating, and weight loss [3]. However, the disease can also present with a wide range of extraintestinal manifestations, including fatigue, migraines, brain fog, dermatitis herpetiformis, dental enamel defects, peripheral neuropathy, cerebellar ataxia, epilepsy, anxiety, and depression [4,5]. Recent studies highlight that CD profoundly affects patients’ lives across multiple dimensions, including physical, emotional and social well-being, and sexual health, which are important determinants of quality of life [6,7].

Currently, the only available treatment is lifelong adherence to a gluten-free diet (*GFD*) [1]. Strict adherence is essential for achieving intestinal healing and alleviating symptoms. However, maintaining a GFD can be challenging due to the ubiquity of gluten, the risk of cross-contamination, inadequate food labeling, and social constraints [8,9]. Several studies have revealed that a considerable percentage of celiac patients do not adhere to a GFD [10]. Rates for strict adherence range from 45% to 90%, depending on the definition and assessment methods used [11]. Low adherence exposes the patient to an increased risk of complications such as malnutrition, anemia, osteoporosis, infertility, intestinal lymphoma, and is linked to impaired health-related quality of life (HRQoL) [6,12].

Reliable tools to evaluate dietary adherence are essential for effective patient management in CD. The Celiac Disease Adherence Test (CDAT), developed by Leffler et al. (2009), is a widely used method for assessing GFD adherence in both research and clinical settings [13,14,15]. In the absence of a standardized instrument for evaluating GFD adherence in Greece, this study primarily aimed to develop and validate a Hellenic version of CDAT (H-CDAT) to enable the accurate assessment of dietary adherence among Greek adult celiac patients. Additionally, the study aimed to evaluate HRQoL, which has not been systematically studied in this population, and to examine the impact of dietary adherence on its various dimensions.

## 2. Materials and Methods

This was a cross-sectional study conducted to translate and validate the Hellenic version of CDAT and to examine the associations between dietary adherence and HRQoL. The study adhered to the ethical principles of the Declaration of Helsinki and received approval from the Institutional Review Board of the Hellenic Mediterranean University in 2024 (Approval Number: 30922). All participants provided informed consent electronically, and data were collected anonymously to ensure confidentiality.

### 2.1. Translation and Adaptation of Questionnaire

The original CDAT was translated and adapted using the Forward-Backward translation method. Initially, permission to translate and validate CDAT into the Hellenic language was obtained via email from Dr. D. A. Leffler. Subsequently, three bilingual translators independently translated the questionnaire into Hellenic (forward translation). The three versions were reviewed by a panel of five dietitians to select the most appropriate Hellenic version of the questionnaire. This selected version, H-CDAT, was then back-translated into English by an independent native English speaker and sent to Dr. Leffler for confirmation.

After receiving confirmation, a pilot study was conducted with 14 CD patients (12 females and 2 males) to assess the clarity, comprehensibility, and cultural appropriateness of H-CDAT. Additionally, the same patients answered the questions: ‘How compliant are you in following a GFD?’ and ‘How difficult is it to follow a GFD?’. The available response options for self-perceived adherence were ‘poor’ (scored as 1), ‘moderate’ (scored as 2), and ‘good’ (scored as 3). Patients characterized the difficulty in following a GFD as low (scored as 1), moderate (scored as 2), and high (scored as 3). The responses to these questions, along with the H-CDAT results, were subjected to statistical analysis as an initial part of the validation process, which indicated that no further modifications were required.

### 2.2. Survey Description and Distribution

The final questionnaire consisted of four sections: demographics, disease- and GFD-related information, H-CDAT, and HRQoL. It was distributed online through platforms such as the Hellenic Coeliac Society, Facebook support groups, hospitals, and clinics. Participants accessed the survey via Google Forms, where the first page included an information sheet summarizing the study’s purpose, objectives, and eligibility criteria. Consent was obtained electronically by requiring participants to check a box before proceeding to the survey questions. The survey was available from 8 November 2024 to 30 November 2024. Participation was voluntary and anonymous, adhering to minimal-risk ethical guidelines. Eligible participants were as follows:

(a) Individuals with CD;
(b) Adults aged ≥ 18 years;
(c) Diagnosed at least one year prior to the study.

Exclusion criteria included individuals with chronic conditions such as cancer, Alzheimer’s disease, chronic renal disease, chronic lung disease, cardiovascular disease, etc.

A total of 110 CD patients responded to the online survey, of which 102, aged 18 to 67 years, met the eligibility criteria and were enrolled in the study.

### 2.3. GFD Adherence

CDAT is a valid self-report measure of GFD adherence [13]. It was translated into the Spanish and Persian languages with appropriate psychometric properties [16,17]. CDAT consists of 7 questions on a 5-point Likert scale, assessing CD symptoms, self-efficacy, reasons for keeping a GFD (motives), and perceived adherence. It uses an additive scoring system with a range from 7 to 35.

Patients completed H-CDAT, which retained the original 1–5 scoring system proposed by Dr. Leffler. As with the original version, scores < 13 indicate Good adherence, scores between 13 and 17 indicate Moderate adherence, and scores > 17 indicate Poor adherence.

### 2.4. Health-Related Quality of Life

Quality of life was assessed using the Short Form Health Survey (SF-36), validated for the Greek population [18,19]. SF-36 is a self-reported scale consisting of 36 questions that cover 8 domains of health: physical functioning (PF), role-physical (RP), bodily pain (BP), general health (GH), vitality (VT), social functioning (SF), role-emotional (RE), and mental health (MH). Each domain score ranges from 0 (poorest health status) to 100 (best health status). PF, RP, and BP are dimensions of physical health, whereas SF, RE, and MH are dimensions of mental health. GH and VT reflect both physical and mental health.

### 2.5. Validation of H-CDAT

To assess internal consistency of the H-CDAT questionnaire, Cronbach’s alpha was calculated based on responses to each of the 7 questions. Additionally, precision was established by examining score distribution for ceiling and floor effects, with values lower than 20% considered acceptable. Factorial construct validity was assessed through Principal Component Analysis (PCA) with varimax rotation and Kaiser normalization. The Kaiser–Meyer–Olkin (KMO) measure of sampling adequacy and Bartlett’s test of sphericity were employed to determine whether the data were suitable for factor analysis.

Due to the lack of equivalent tests, the SF-36 questionnaire was administered alongside the H-CDAT and diet-related questions to examine concurrent validity through correlations between H-CDAT items, the SF-36 domains, and diet-related issues.

### 2.6. Statistical Analysis

For statistical analysis, the Statistical Package for the Social Sciences (SPSS), version 29, was used. Nominal and ordinal data were summarized as absolute frequencies and percentages, while continuous variables were summarized as mean and standard deviation. Categorical variables were compared with the chi square test. The level of significance was *p* < 0.05. Quantitative variables were compared with the Student’s *t*-test in the case of a normal distribution or otherwise with the Mann–Whitney U and Kruskal–Wallis nonparametric tests. ANOVA was used to compare means across multiple groups. The Independent Samples *t*-Test was used to compare the mean SF-36 domain scores between patients and the general Greek population [19]. Additionally, Pearson and Spearman tests were used to examine correlations between continuous variables with normal and non-normal distribution. The normal distribution fit was studied using the Kolmogorov–Smirnov test. A factorial analysis was conducted using PCA and varimax with Kaiser normalization.

## 3. Results

### 3.1. Patient Characteristics

The study included 102 patients, 85 females, and 17 males. The mean age was 40.74 ± 9.58 years for females and 43.59 ± 11.77 years for males. The mean age at diagnosis was 34.36 ± 11.91 years for females and 36.41 ± 13.49 years for males. Patients were on a GFD for 7.1 ± 7.0 years, ranging from 1 to 36 years. The descriptive statistics of sociodemographic characteristics of the patients are summarized in Table 1.

The most common symptoms reported prior to diagnosis included bloating (69.6%), fatigue (62.7%), and abdominal pain (51%), whereas associated autoimmune disorders included Hashimoto’s thyroiditis (23.5%) and inflammatory bowel disease (IBD, 3.9%), as detailed in Table 2. The majority of patients (76.5%) reported good self-perceived adherence to the GFD (Table 3). However, over half of the patients (52.9%) reported high difficulty in following the diet. Additionally, 51% of patients had never consulted a dietitian for guidance on managing their GFD. In terms of symptom resolution with the GFD, 51% of patients reported full resolution of symptoms, while 34.3% reported only partial resolution.

### 3.2. H-CDAT Validation

Upon analyzing the relevance of the factorial analysis, we found a KMO value of 0.605, which exceeds the minimum threshold of 0.5 recommended by Taherdoost [20], indicating that our sample is adequate for running a factor analysis. We also found a significant Bartlett sphericity test, with *p* < 0.001. The original CDAT questionnaire and its Hellenic validated version, H-CDAT, are shown in Table 4. H-CDAT retains the core components of CDAT while ensuring cultural and linguistic relevance for Hellenic populations. PCA with varimax rotation suggested three factors that account for 70.613% of the variance (Table 5). The first factor, (symptoms), included questions 1 and 2, the second factor (motivation, self-efficacy, and mood), included questions 3, 4, and 5 and the third one (reasons for keeping a GFD, perceived adherence to the GFD) included questions 6 and 7. The feasibility study showed that 100% of the participants answered all the questions. The floor effect was 3.9% and the ceiling effect was 0%. An analysis of the internal consistency of each factor to the adaptation returned Cronbach alpha coefficients of 0.712, 0.617, and 0.533, respectively.

Regarding concurrent validity, the lack of equivalent tests led us to examine correlations between H-CDAT questions and self-perceived GFD adherence, the degree of difficulty in following a GFD, and HRQoL SF-36 domains (shown in Table 6). Question 1 correlated with MH (r = −0.587; *p* < 0.001) and VT (r = −0.733; *p* < 0.001); and question 2 with GH (r = −0.260; *p* = 0.008) and BP (r = −0.333; *p* < 0.001). Question 3 correlated with SF (r = −0.415; *p* < 0.001) and RE (r = −0.298; *p* = 0.002). Question 3 also correlated with the degree of difficulty in following a GFD (r = 0.353; *p* < 0.001), indicating that patients who found the GFD more difficult to follow were less able to adhere to the diet when dining out. Questions 4 and 5 correlated with MH (r = −0.310, *p* = 0.002; r = −0.218, *p* = 0.028, respectively). Question 5 also correlated with SF (r = −0.217; *p* = 0.029). Question 6 was significantly associated with self-perceived GFD adherence (r = −0.221; *p* = 0.026), suggesting that patients who considered accidental gluten exposures less important to their health (indicated by a higher score) had a lower score of self-perceived adherence (indicating poor adherence). Question 7 correlated with GH (r = −0.205; *p* = 0.038) and self-perceived adherence (r = −0.614; *p* < 0.001), suggesting that patients who have eaten gluten-containing foods on purpose more times over the past 4 weeks (indicated by a higher score) had lower GH and lower self-perceived adherence.

### 3.3. HRQoL Status

The patients’ mean SF-36 scores across all HRQoL domains are depicted in Table 6. Also, the respective values of the general Greek population, obtained by Pappa et al. (2005), are shown alongside the *p*-values [19]. Patients exhibited significantly lower HRQoL scores in the domains of GH, MH, SF, VT, RE, and RP compared to the general Greek population. No statistically significant gender differences among the patients were observed across the domains, except for VT levels, where males scored significantly higher (66.18 ± 23.35) than females (51.29 ± 21.69, *p* = 0.023).

Furthermore, a subgroup of CD patients (*n* = 4, 3.9%) had concurrent IBD. Marked differences were observed in PF and RP between the IBD subgroup (*n* = 4) and the remaining CD patients (*n* = 98). For PF, the IBD subgroup had a mean score of 77.5 ± 26.30, which was lower than the mean score of 80.51 ± 25.61 in the remaining CD patients. For RP, the IBD subgroup had a mean score of 50.00 ± 57.35, which was considerably lower than the mean score of 70.15 ± 37.07 in the other CD patients. However, statistical analysis using the Mann–Whitney U test revealed that the differences did not reach statistical significance (*p* = 0.922 for PF and *p* = 0.442 for RP), most likely due to the small sample size of IBD patients.

### 3.4. H-CDAT Dietary Adherence

According to H-CDAT, Good adherence was observed in 38.2% of patients, Moderate adherence in 42.2%, and Poor adherence in 19.6% (Table 7). These results were notably lower than the self-perceived rates reported earlier by the patients (76.5% good, 23.5% moderate, 0% poor).

H-CDAT scores correlated with both self-perceived adherence (r = −0.573, *p* < 0.001) and the degree of difficulty in following a GFD (r = 0.432, *p* < 0.001). H-CDAT scores also correlated significantly with 7 of the 8 domains of HRQoL: GH (r = −0.400, *p* < 0.001), MH (r = −0.495, *p* < 0.001), VT (r = −0.409, *p* < 0.001), BP (r = −0.307, *p* = 0.005), RP (r = −0.223, *p* = 0.043), RE (r= −0.290, *p* = 0.008), and SF (r = −0.423, *p* < 0.001).

### 3.5. HRQoL Stratified by H-CDAT Adherence

**Figure 1:**
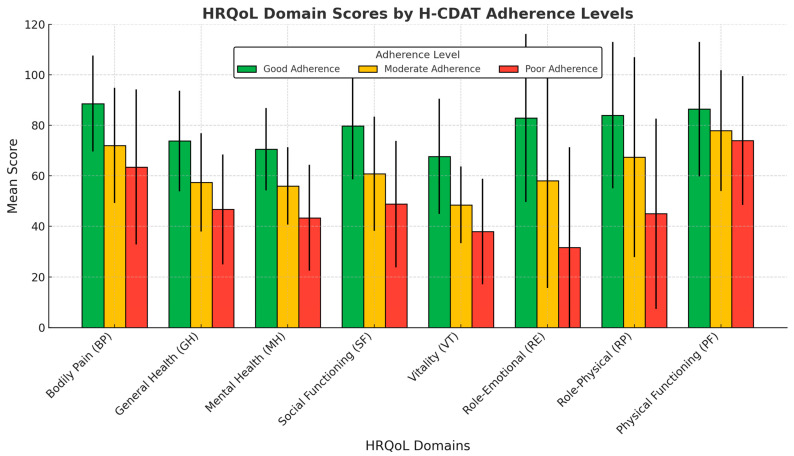
HRQoL domain scores by H-CDAT adherence levels in patients. Good adherence (*n* = 39), Moderate adherence (*n* = 43), Poor adherence (*n* = 20). Error bars represent standard deviation (SD) for each group. HRQoL, health-related quality of life; H-CDAT, Hellenic celiac dietary adherence test.

To assess the impact of dietary adherence on HRQoL, patients were stratified into three adherence groups based on their H-CDAT scores: Good (*n* = 39), Moderate (*n* = 43), and Poor (*n* = 20). The mean HRQoL scores for each group across the eight SF-36 domains are presented in Figure 1. Patients with Good adherence consistently demonstrated higher HRQoL scores compared to those with Moderate and Poor adherence, with significant differences observed across both physical and mental health domains.

In the BP domain, scores were 88.6 ± 19.0, 72.0 ± 22.8, and 63.5 ± 30.7 for the Good, Moderate, and Poor adherence groups, respectively (*p* < 0.001 for Good vs. Moderate and Good vs. Poor; *p* = 0.311 for Moderate vs. Poor). For GH, the scores were 73.8 ± 19.9, 57.4 ± 19.5, and 46.7 ± 21.7, respectively (*p* < 0.001 for Good vs. Moderate and Good vs. Poor; *p* = 0.096 for Moderate vs. Poor). In the MH domain, scores were 70.6 ± 16.3, 56.0 ± 15.3, and 43.4 ± 21.0 (*p* < 0.001 for Good vs. Moderate and Good vs. Poor; *p* = 0.021 for Moderate vs. Poor). Similarly, SF scores were 79.8 ± 21.2, 60.8 ± 22.6, and 48.8 ± 25.0 (*p* < 0.001 for Good vs. Moderate and Good vs. Poor; *p* = 0.155 for Moderate vs. Poor).

For the VT domain, scores were 67.7 ± 22.8, 48.5 ± 15.2, and 38.0 ± 20.9 (*p* < 0.001 for Good vs. Moderate and Good vs. Poor; *p* = 0.149 for Moderate vs. Poor). In RE, the scores were 82.9 ± 33.2, 58.1 ± 42.5, and 31.7 ± 39.7 (*p* = 0.005 for Good vs. Moderate; *p* = 0.025 for Moderate vs. Poor; *p* < 0.001 for Good vs. Poor). In the RP domain, scores were 84.0 ± 29.0, 67.4 ± 39.5, and 45.0 ± 37.7 (*p* = 0.051 for Good vs. Moderate; *p* = 0.046 for Moderate vs. Poor; *p* < 0.001 for Good vs. Poor). Lastly, in the PF domain, scores were 86.4 ± 26.6, 77.9 ± 23.9, and 74.0 ± 25.5 (*p* = 0.011 for Good vs. Moderate; *p* = 0.697 for Moderate vs. Poor; *p* = 0.042 for Good vs. Poor).

## 4. Discussion

In the absence of a practical, validated tool for evaluating GFD adherence among CD patients in Greece, the primary aim of this study was to develop and validate a Hellenic version of the original CDAT, which was created by gastroenterologist Dr. Leffler [13]. The resulting H-CDAT exhibited good psychometric properties and internal consistency. Factor analysis revealed that H-CDAT comprised three factors, as is consistent with the structure of both the original and Persian versions of the questionnaire [13,17]. Due to the lack of equivalent tests, H-CDAT was further validated through correlations between each H-CDAT question and HRQoL domains, as well as self-perceived GFD adherence and the degree of difficulty in following a GFD. The results confirmed that H-CDAT is a reliable tool for assessing adherence to the GFD in adult Hellenic CD patients.

Based on H-CDAT, only 38.2% of the patients demonstrated good adherence. Although H-CDAT scores correlated significantly with the patients’ self-perceived adherence scores, the actual level of dietary adherence was significantly lower than their perceived adherence. This discrepancy indicates that patients are overestimating their adherence, possibly due to unintentional gluten consumption or a misunderstanding of the dietary guidelines concerning a strict GFD. As a result, the study population exhibited lower adherence rates compared to the 45% to 90% range reported in the literature [11], highlighting the need for improved education to promote better adherence.

Failure to maintain strict dietary adherence can lead to persistent symptoms and villous atrophy, as well as a significantly increased risk of long-term health complications and mortality [2,21]. Additionally, CD was linked to a higher risk of secondary autoimmune diseases, such as type 1 diabetes and Hashimoto’s thyroiditis, though it remains unclear whether strict adherence to a GFD can prevent or ameliorate secondary autoimmunity [1,22]. Given the association between CD and endocrine autoimmunity, patients with CD should be screened for type 1 diabetes and/or autoimmune thyroid disease [23].

Despite the serious health risks, maintaining strict adherence to a GFD is extremely challenging. Previous studies have identified several barriers that impact the ability to maintain a GFD, including the high cost and limited availability of gluten-free foods, especially in restaurants and social settings, the risk of cross-contamination, and a general lack of knowledge [9,24]. In fact, dining out was identified as a significant challenge for patients in this study, strongly correlating with the difficulty in following a GFD. The strong association between H-CDAT adherence and the degree of difficulty in following a GFD reveals that adherence is significantly hindered by these barriers.

Notably, the majority of patients in this study found the GFD very difficult to follow and had never consulted a dietitian for guidance. This lack of professional dietary support may explain the overestimation of adherence observed in the current study and highlights the critical need for dietitians with expertise in CD management. Dietitian consultation and follow-up are essential for accurate GFD education, understanding food labeling, addressing cross-contamination risks, and preventing complications from an unbalanced GFD, such as nutritional deficiencies and metabolic disorders [9,25].

In addition to exploring dietary adherence, the study also aimed to assess HRQoL, which, to the best of our knowledge, has never been evaluated in Greek adult CD patients. HRQoL refers to a person’s perceived well-being in the physical, mental, and social aspects of health, as well as their ability to function in daily activities [26]. It is a key health indicator used to evaluate the impact of disease and treatment on patients’ daily lives and overall functioning [27].

According to the SF-36 results, patients scored significantly lower in most HRQoL domains compared to the general Greek population [19]. Specifically, significant reductions were observed in GH, MH, SF, VT, and role activities impacted by physical and emotional problems. These findings align with those of Dochat et al. (2024), who reported that CD patients often experience diminished general health, social functioning, and mental well-being [28]. Similarly, Al-Qefari and colleagues (2018) found poor scores across all HRQoL subscales in CD patients, with emotional well-being, social functioning, and role-emotional being the most severely affected [29].

Although patients scored lower in the RP domain, PF was not significantly impacted. This suggests that while patients may retain the physical capacity to perform basic physical activities, they experience greater difficulty in completing physically demanding daily tasks (such as work duties, household chores, or sustained physical activities) due to their physical health issues. Interestingly, patients with concurrent IBD exhibited lower scores in both PF and RP compared to other CD patients. Although these differences were not statistically significant, likely due to the small number of IBD patients, this trend may hold important clinical relevance, as IBD alone was shown to impair physical functioning due to chronic inflammation and disease-related symptoms [30,31].

Moreover, the study revealed strong negative correlations between H-CDAT scores and 7 of the 8 HRQoL dimensions, including GH, MH, VT, BP, SF, RP, and RE, highlighting the beneficial impact of dietary adherence on both physical and mental health. Importantly, stronger adherence to a GFD was associated with lower levels of bodily pain and fewer limitations in performing physically demanding daily tasks, suggesting that effective dietary management may alleviate physical discomfort and improve the ability to engage in daily physical activities. These findings align with studies supporting the positive effects of a GFD on the HRQoL of CD patients, despite the psychosocial challenges associated with the diet [6,32,33]. Improved adherence to a GFD is strongly linked to better symptom management in CD, which significantly enhances patients’ quality of life [34].

Consequently, when patients were stratified according to H-CDAT adherence levels, marked differences in multiple HRQoL domains were observed. Both the Kruskal–Wallis test and ANOVA results strongly suggested that better dietary adherence, as measured by H-CDAT, is associated with significantly higher scores across all HRQoL domains. More specifically, substantial differences were observed between patients with good and poor adherence, which were highly significant in nearly all HRQoL domains. In contrast, the differences between moderate and poor adherence were less pronounced, with statistically significant differences observed only in the MH and RP domains.

Most importantly, the comparison between good and moderate adherence revealed striking differences across nearly all HRQoL domains, emphasizing that moderate adherence is insufficient for optimal outcomes. The RP domain, which measures how physical health issues affect daily responsibilities, was the only exception, showing a borderline insignificant difference. This may reflect the subjective nature of the RP domain, making it harder to capture the impact of intermediate adherence levels. Nevertheless, overall adherence significantly affected the RP domain, with strict adherence leading to the greatest improvements. Aligning with our findings, a meta-analysis by Rustagi et al. (2020) showed that patients with partial GFD adherence had lower HRQoL compared to those with strict adherence [35]. This underscores the critical role of strict dietary adherence in improving health outcomes in CD patients and highlights the importance of dietetic intervention to achieve optimal patient outcomes.

The present study has several limitations that should be acknowledged. First, it did not include a test–retest assessment to further evaluate the construct reliability of H-CDAT. Second, the study lacked a control group, which would have allowed for direct comparisons of HRQoL scores between patients and matched healthy individuals. Another limitation was the high preponderance of female patients in the study group, which was markedly higher than the typical female-to-male ratio reported in Europe but consistent with previous findings from Greece [9]. However, given the scarcity of nationwide data on celiac disease in Greece and the limited information on gender ratios, the representativeness of these findings cannot be conclusively established. Additionally, as an online survey, it was subject to sample/selection bias and relied solely on self-reported data. Therefore, unlike the study by Nikniaz et al. (2020), this study did not analyze the correlation between H-CDAT scores and anti-tissue transglutaminase immunoglobulin A titers [17]. Nevertheless, the high correlation found in the original version by Leffler et al. strengthens the convergent validity of H-CDAT for evaluating dietary adherence in celiac patients [13]. Furthermore, the findings of this study provide valuable insights into the relationship between dietary adherence and HRQoL. The strong correlations observed confirm that H-CDAT is a reliable tool for assessing GFD adherence in adult Hellenic CD patients. Longitudinal studies, ideally with a more representative gender ratio, are warranted to further evaluate the impact of GFD adherence on health outcomes in this population and to enhance the generalizability of these findings.

## 5. Conclusions

The present study revealed inadequate GFD adherence in most celiac patients, despite their perception of effective adherence. It also highlighted low HRQoL scores across most subscales, and a lack of dietitian consultations and follow-up. Stratification by H-CDAT adherence levels showed significant differences across all HRQoL dimensions, underscoring the critical role of strict GFD adherence in improving overall health. These findings emphasize the importance of dietetic intervention to ensure optimal patient outcomes.

## Figures and Tables

**Table 1:** Descriptive statistics of sociodemographic characteristics of patients.

Variable	Frequency Percentage (%)
**Gender**	
Female	85 (83.3)
Male	17 (16.7)
**Education Level**	
Secondary Education	22 (21.6)
Bachelor	44 (43.1)
Master–Ph.D.	26 (25.5)
Other	10 (9.8)
**Civil Marital status**	
Single	29 (28.4)
Married	62 (60.8)
Other	11 (10.8)
**Employment status**	
Unemployed	20 (19.6)
Employed	78 (76.5)
Retired	4 (3.9)

**Table 2:** Most common symptoms prior to diagnosis and associated autoimmune disorders.

Symptom/Disorder	Total (*n* = 102) Frequency Percentage (%)	Female (*n* = 85) Frequency Percentage (%)	Male (*n* = 17) Frequency Percentage (%)
**Bloating**	71 (69.6)	63 (74.1)	8 (47.1)
**Fatigue**	64 (62.7)	58 (68.2)	6 (35.3)
**Abdominal pain**	52 (51.0)	47 (55.3)	5 (29.4)
**Diarrhea**	49 (48.0)	42 (49.4)	7 (41.2)
**Weight loss**	33 (32.4)	28 (32.9)	5 (29.4)
**Brain fog**	32 (31.4)	30 (35.3)	2 (11.8)
**Constipation**	30 (29.4)	27 (31.8)	3 (17.6)
**Depression**	27 (26.5)	25 (29.4)	2 (11.8)
**Migraine**	23 (22.5)	21 (24.7)	2 (11.8)
**Muscle cramps**	22 (21.6)	21 (24.7)	1 (6.9)
**Vomiting**	20 (19.6)	18 (21.2)	2 (1.8)
**Skin rashes**	20 (19.6)	17 (20.0)	3 (17.6)
**Iron deficiency anemia**	11 (10.8)	8 (9.4)	3 (17.6)
**Hashimoto’s thyroiditis**	24 (23.5)	20 (23.5)	4 (23.5)
**Inflammatory bowel disease**	4 (3.9)	4 (4.7)	0 (0)
**Psoriasis**	2 (2.0)	1 (1.2)	1 (5.9)
**Systemic *lupus* erythematosus**	2 (2.0)	1 (1.2)	1 (5.9)

**Table 3:** Self-perceived GFD adherence, difficulty, dietitian visits, and symptom resolution.

Variable	Frequency (%)	Number of Patients (*n*)
**Self-perceived GFD adherence**		
Good adherence	76.50	78
Moderate adherence	23.50	24
**Difficulty in following GFD**		
High difficulty	52.90	54
Moderate difficulty	35.30	36
Low difficulty	11.80	12
**Dietitian visits**		
Never visited a dietitian	51	52
Visited once	35.30	36
Visited more than once	13.70	14
**Symptom resolution**		
Full symptom resolution with GFD	51	52
Partial symptom resolution with GFD	34.30	35
No symptoms prior to diagnosis	6.90	7
Symptoms remained despite GFD	7.80	8

**Table 4:** CDAT and H-CDAT questionnaire.

Question			Score				
	CDAT	H-CDAT	1	2	3	4	5
**1**	Have you been bothered by low energy level during the past 4 weeks?	*Έχετε νιώσει τις τελευταίες 4 εβδομάδες να έχετε χαμηλή ενέργεια;*	*Ποτέ*	*Λίγες φορές*	*Κάποιες φορές*	*Πολλές φορές*	*Συνέχεια*
**2**	Have you been bothered by headaches during the past 4 weeks?	*Σας έχουν ενοχλήσει πονοκέφαλοι τις τελευταίες 4 εβδομάδες;*	*Ποτέ*	*Λίγες φορές*	*Κάποιες φορές*	*Πολλές φορές*	*Συνέχεια*
**3**	I am able to follow a GFD when dining outside my home	*Μπορώ να ακολουθήσω δίαιτα ελεύθερη γλουτένης όταν γευματίζω εκτός σπιτιού*	*Συμφωνώ απόλυτα*	*Συμφωνώ κάπως*	*Oύτε συμφωνώ, ούτε διαφωνώ*	*Διαφωνώ κάπως*	*Διαφωνώ απόλυτα*
**4**	Before I do something I carefully consider the consequences	*Πριν κάνω κάτι σκέφτομαι προσεκτικά τις συνέπειες*	*Συμφωνώ απόλυτα*	*Συμφωνώ κάπως*	*Oύτε συμφωνώ, ούτε διαφωνώ*	*Διαφωνώ κάπως*	*Διαφωνώ απόλυτα*
**5**	I do not consider myself a failure	*Δεν θεωρώ τον εαυτό μου αποτυχημένο*	*Συμφωνώ απόλυτα*	*Συμφωνώ κάπως*	*Oύτε συμφωνώ,ούτε διαφωνώ*	*Διαφωνώ κάπως*	*Διαφωνώ απόλυτα*
**6**	How important to your health are accidental gluten exposures?	*Πόσο σημαντικές για την υγεία σας είναι οι τυχαίες εκθέσεις στη γλουτένη;*	*Πολύ σημαντικές*	*Κάπως σημαντικές*	*Δεν με απασχολεί/Δεν είμαι σίγουρος/η*	*Λίγο σημαντικές*	*Καθόλου σημαντικές*
**7**	Over the past 4 weeks, how many times have you eaten foods containing gluten on purpose?	*Τις τελευταίες 4 εβδομάδες, πόσες φορές έχετε καταναλώσει εν γνώσει σας τροφές που περιέχουν γλουτένη;*	0 *(ποτέ)*	1–2	3–5	6–10	>10

CDAT: celiac dietary adherence test; H-CDAT: Hellenic celiac dietary adherence test; GFD: gluten-free diet.

**Table 5:** Factor analysis of H-CDAT.

Question	Component		
	1	2	3
**1**	0.177	**0.841**	0.191
**2**	−0.024	**0.881**	−0.031
**3**	**0.729**	0.297	−0.205
**4**	**0.652**	0.214	0.394
**5**	**0.825**	−0.180	0.100
**6**	−0.150	0.112	**0.839**
**7**	0.270	−0.004	**0.753**

H-CDAT: Hellenic celiac dietary adherence test. Method of extraction: Principal Component Analysis; rotation method: varimax with Kaiser normalization. Rotation converged in 4 iterations.

**Table 6:** HRQoL domain scores of CD patients and general Greek population [19].

Domain	CD Patients *n* = 102	General Greek Population *n* = 1426	*p*-Value
**Bodily Pain (BP)** *Evaluates the intensity of pain and its interference with overall functioning. Lower scores indicate more severe pain*	76.67 ± 25.06	72.98 ± 31.66	0.164
**General Health (GH)** *Assesses overall health perception*	61.62 ± 22.46	67.46 ± 23.54	0.013 *
**Mental Health (MH)** *Assesses psychological distress and emotional well-being*	59.10 ± 19.59	68.23 ± 21.26	<0.001 *
**Social Functioning (SF)** *Assesses limitations in social activities due to physical or emotional problems*	65.69 ± 25.35	82.05 ± 28.12	<0.001 *
**Vitality (VT)** *Measures overall energy and fatigue levels*	53.77 ± 22.55	66.53 ± 22.39	<0.001 *
**Role-Emotional (RE)** *Measures role limitations due to emotional problems*	62.42 ± 42.65	81.53 ± 36.31	<0.001 *
**Role-Physical (RP)** *Measures limitations in usual role activities due to physical health problems*	69.36 ± 37.87	79.74 ± 37.72	0.009 *
**Physical Functioning (PF)** *Evaluates the ability to perform physical activities*	80.39 ± 25.51	80.76 ± 25.62	0.888

Values for general Greek population were obtained from Pappa et al. (2005) [19]. Values are expressed as mean ± SD. HRQoL: health-related quality of life; CD: celiac disease. * Significant differences between CD patients and general Greek population.

**Table 7:** Dietary adherence scores and categories by gender according to H-CDAT.

Dietary Adherence Scores (Mean ± SD)	Total *n* = 102 13.96 ± 4.05	Female *n* = 85 14.15 ± 4.09	Male *n* = 17 13.00 ± 3.84
Dietary Adherence Category	*n* (%)	*n* (%)	*n* (%)
***Good adherence*** (H-CDAT score <13)	39 (38.2)	32 (37.6)	7 (41.2)
***Moderate adherence*** (H-CDAT score 13–17)	43 (42.2)	36 (42.4)	7 (41.2)
***Poor adherence*** (H-CDAT score >17)	20 (19.6)	17 (20)	3 (17.6)

H-CDAT: Hellenic celiac dietary adherence test.

## Data Availability

The data supporting the findings of this study are included in the article, further inquiries can be directed to the corresponding author.
